# Molecular characterizations of *Cryptosporidium* spp. and *Enterocytozoon bieneusi* in brown rats (*Rattus norvegicus*) from Heilongjiang Province, China

**DOI:** 10.1186/s13071-018-2892-7

**Published:** 2018-05-24

**Authors:** Wei Zhao, Jianguang Wang, Guangxu Ren, Ziyin Yang, Fengkun Yang, Weizhe Zhang, Yingchu Xu, Aiqin Liu, Hong Ling

**Affiliations:** 1 Department of Parasitology, Harbin Medical University China, Harbin, 150081 Heilongjiang China; 2 Department of Microbiology, Wu Lien-Teh Institute, Harbin Medical University, Heilongjiang Provincial Key Laboratory of Infection and Immunity, Key Laboratory of Pathogen Biology, Harbin, 150081 China; 3 Department of Immunology, Harbin Medical University, Harbin, 150081 China

**Keywords:** *Cryptosporidium*, *Enterocytozoon bieneusi*, Zoonotic, Brown rats, Genotyping

## Abstract

**Background:**

*Cryptosporidium* spp. and *Enterocytozoon bieneusi* are prevalent zoonotic pathogens responsible for the high burden of diarrheal diseases worldwide. Rodents are globally overpopulated and are known as reservoirs or carriers of a variety of zoonotic pathogens including *Cryptosporidium* spp. and *E. bieneusi*. However, few data are available on genetic characterizations of both pathogens in rodents in China. The aim of the present work was to determine the prevalence and genetic characterizations of *Cryptosporidium* spp. and *E. bieneusi* in brown rats (*Rattus norvegicus*) from Heilongjiang, China.

**Methods:**

A total of 242 wild brown rats were captured in Heilongjiang Province of China. A fresh fecal specimen was collected directly from the intestinal and rectal content of each brown rat. All the fecal specimens were examined for the presence of *Cryptosporidium* spp. and *E. bieneusi* by PCR and sequencing of the partial small subunit (SSU) rRNA gene and the internal transcribed spacer (ITS) region of the rRNA gene of the two pathogens, respectively.

**Results:**

The infection rate was 9.1% (22/242) for *Cryptosporidium* spp. and 7.9% (19/242) for *E. bieneusi*. Sequence analysis confirmed the presence of *C. ubiquitum* (1/22, 4.5%) and three genotypes of *Cryptosporidium*, including *Cryptosporidium* rat genotype I (14/22, 63.6%), *Cryptosporidium* rat genotype IV (6/22, 27.3%) and *Cryptosporidium suis-*like genotype (1/22, 4.5%). Meanwhile, two *E. bieneusi* genotypes were identified, including D (17/19, 89.5%) and Peru6 (2/19, 10.5%).

**Conclusions:**

To the best of our knowledge, *Enterocytozoon bieneusi* genotype Peru6 was identified in rodents for the first time globally and *Cryptosporidium* rat genotype I and *Cryptosporidium* rat genotype IV were found in rats in China for the first time. The finding of zoonotic *C. ubiquitum* and *C. suis*-like genotype, as well as *E. bieneusi* genotypes, suggests that brown rats pose a threat to human health. It is necessary to control brown rat population in the investigated areas and improve local people’s awareness of the transmission risk of the two pathogens from brown rats to humans.

## Background

*Cryptosporidium* spp. and *Enterocytozoon bieneusi* are two common opportunistic pathogens in humans and have been reported to be associated with diarrhea [1, 2]. However, clinical symptoms of the infection are variable depending on the health status of the infected hosts, displaying asymptomatic infection or self-limiting diarrhea in healthy people, and chronic or life-threatening diarrhea in immunocompromised individuals [2, 3]. In addition to humans, numerous animal species are also the hosts of the two pathogens, suggesting a zoonotic nature of the two parasitic diseases [4, 5]. Meanwhile, they have also been identified in some water bodies and food products, indicating the possibility of water-borne and food-borne transmission [3, 6, 7]. Because of the clinical and public health importance of *Cryptosporidium* spp. and *E. bieneusi*, both have been ranked on category B list, in which the pathogens are defined as the second highest priority organisms/biological agents by the National Institutes of Health (NIH) of the USA [8]. Meanwhile, *Cryptosporidium* spp. is ranked fifth among the 24 most important food-borne parasites in a global ranking by a joint Food and Agriculture Organization (FAO)/World Health Organization (WHO) expert committee [9]. *Enterocytozoon bieneusi* is listed on the Environmental Protection Agency (EPA) microbial contaminant candidate list of concern for waterborne transmission [10].

*Cryptosporidium* is a complex genus. To date, at least 31 species and more than 40 genotypes of *Cryptosporidium* have been identified by sequencing the small subunit (SSU) rRNA gene [2, 11]. Of them, 21 *Cryptosporidium* species/genotypes have been isolated in humans and *C. parvum* is generally considered to be zoonotically transmitted [2]. Some zoonotic outbreaks of cryptosporidiosis caused by *C. parvum* have been reported and confirmed at a subtype level, such as calf-derived IIaA15G2R1 in the UK [12] and sheep-derived IIaA17G1R1, IIaA15G2R1 in the UK and IIaA20G2R1 in Italy [12, 13]. Thus, molecular epidemiological investigations of animal cryptosporidiosis have been paid more attention. However, most of them focus on farm animals (pigs, sheep and cattle) or pets (dogs and cats) [5, 14]. Rodents, as the most widespread groups of mammals, have been reported to carry at least 11 species and more than 20 genotypes of *Cryptosporidium* as vectors or reservoirs, including *C. parvum*, *C. muris*, *C. ubiquitum*, *C. meleagridis*, *C. scrofaru*m, *C. wrairi*, *C. tyzzeri*, *C. rubeyi*, *C. andersoni*, *C. hominis*, *C. suis* and rat genotypes (I-IV), mouse genotypes (II, III) and the Naruko genotype, ferret genotype, chipmunk genotypes (I, II), skunk genotype, hamster genotype, deer mouse genotypes (I-IV), vole genotype, bear genotype, muskrat genotypes (I, II) and ground squirrel genotypes (I-III) [15–38]. Among them, *Cryptosporidium* species except for *C. rubeyi* and two genotypes (chipmunk genotype I and skunk genotype) have also been found in humans [11].

For *E. bieneusi*, at least 240 genotypes have been identified by analyzing the internal transcribed spacer (ITS) region (~243 bp) of the rRNA gene and they have been classified into nine groups (groups 1-9) by phylogenetic analysis [39, 40]. Group 1 is composed of the common zoonotic genotypes and groups 2-9 mostly contain host-adapted genotypes [11]. To date, more than 70 genotypes have been found in humans, 33 of which are also found in animals, supporting presumption of zoonotic potential [1, 5]. In fact, zoonotic transmission of *E. bieneusi* has been reported in Peru, which occurred between a child and guinea pigs [41]. *Enterocytozoon bieneusi* has been detected in many rodent species and 35 genotypes have been identified, including 12 zoonotic genotypes (BEB6, C, D, EbpA, EbpC, H, Peru8, Peru11, Peru16, PigITS5, S6 and TypeIV) [22, 41–48].

Currently, due to limited effect of nitazoxanide on cryptosporidiosis and fumagillin on microsporidiosis caused by *E. bieneusi* [11], transmission control and prevention of infection of the two pathogens targeting the epidemiologic cycles are the key effective strategies. Understanding *Cryptosporidium* spp. and *E. bieneusi* epidemiology in wide range of hosts, exploring the molecular phylogeny and assessing zoonotic potential of animal-derived isolates are the key steps to prevent and reduce occurrence of the two parasitic diseases. Brown rats (*Rattus norvegicus*) are one of the most common rodent species, and usually live almost everywhere humans are. The dynamic activity of rodents facilitates the transmission and spread of various diseases including cryptosporidiosis and microsporidiosis caused by *E. bieneusi* [49].

In Heilongjiang Province of China, *Cryptosporidium* spp. and *E. bieneusi* are prevalent in a variety of species of animals [50, 51]. Moreover, they have been found in human immunodeficiency virus (HIV)-infected and acquired immunodeficiency syndrome (AIDS)-patients (unpublished data), cancer patients and children [52, 53]. The source of the human infection is still unclear. This study aimed to determine the prevalence of *Cryptosporidium* spp. and *E. bieneusi* in brown rats from various regions of Heilongjiang, China, to characterize the isolates and assess their zoonotic potential.

## Methods

### Study sites and rodent collections

During a three-year period from April 2014 to June 2017, 242 brown rats were captured from five distinct regions of Heilongjiang Province, China, including 55 from a granary in Xingren Town, 30 from a cattle farm in Xingren Town, 73 from a pig farm in Mingshui County, 27 from a pig farm in Qinggang County, 37 from a sheep farm in Baoqing County and 20 from a subdistrict in Harbin City (Table 1). All rats were captured in cage traps baited with sunflower seeds and peanut/sesame butter. In each location, 20 cage traps were installed at sunset and gathered before sunrise, with traps 5 m apart in transects. All rats were transported to the laboratory within 48 h after being captured and were killed by CO_2_ inhalation.

**Table 1:** Prevalence and distribution of *Cryptosporidium* species/genotypes and *E. bieneusi* genotypes in brown rats in Heilongjiang Province of China

Source	Location	No. examined	*Cryptosporidium*		*E. bieneusi*	
			Positive (%)	Species/Genotype (*n*)	Positive (%)	Genotype (*n*)
Farms						
Cattle farm	Xingren	30	2 (6.7)	Rat genotype I (2)	1 (3.3)	D (1)
Pig farm	Mingshui	73	12 (16.4)	Rat genotype I (9); Rat genotype IV (3)	3 (4.1)	D (3)
Pig farm	Qinggan	27	3 (11.1)	Rat genotype I (3)	3 (11.1)	D (3)
Sheep farm	Baoqing	37	1 (2.7)	*C. ubiquitum* (1)	10 (27.0)	D (8); Peru6 (2)
Granary	Xingren	55	4 (7.3)	Rat genotype IV (3); *Suis*-like genotype (1)	1 (1.8)	D (1)
Subdistrict	Harbin	20	0	*–*	1 (5.0)	D (1)
Total		242	22 (9.1)	Rat genotype I (14); Rat genotype IV (6); *Suis*-like genotype (1); *C. ubiquitum* (1)	19 (7.4)	D (17); Peru6 (2)

### Fecal sample collection and DNA extraction

A fresh fecal specimen (approximately 500 mg) was collected directly from the intestinal and rectal content of each brown rat. All specimens were washed with distilled water by centrifugation for 10 min at 1,500× *g* at room temperature. Genomic DNA was extracted directly from approximately 200 mg of each processed specimen using QIAamp DNA Mini Stool Kit (Qiagen, Hilden, Germany) according to the manufacturer’s procedures. The lysis temperature was increased to 95 °C in order to obtain high DNA yield. DNA was eluted in 200 μl of AE elution buffer (provided with the kit) and stored at -20 °C prior to PCR analysis.

### Genotyping of *Cryptosporidium* spp. and *E. bieneusi*

*Cryptosporidium* spp. in the fecal specimens was identified by nested PCR amplification of a SSU rRNA gene fragment of ~830 bp designed by Xiao et al. [54]. Each PCR consisted of 35 cycles of denaturation at 94 °C for 45 s, annealing at 60 °C for 45 s, and extension at 72 °C for 60 s; an initial denaturation step consisting of incubation at 94 °C for 5 min and a final extension step consisting of incubation at 72 °C for 10 min were also included [54]. *Enterocytozoon bieneusi* was identified and genotyped by nested PCR amplification of an approximately 390 bp nucleotide fragment of the rRNA gene, containing 76 bp of the 3' end of the SSU rRNA gene, 243 bp of the ITS region, and 70 bp of the 5' end of the large subunit (LSU) rRNA gene designed by Buckholt et al. [55]. The two sets of cycling parameters were as follows: 35 cycles of 94 °C for 30 s, 57 °C for 30 s, and 72 °C for 40 s and 30 cycles of 94 °C for 30 s, 55 °C for 30 s, and 72 °C for 40 s, with both of them having an initial denaturation step at 94 °C for 5 min and a final extension step at 72 °C for 10 min [55]. TaKaRa Taq DNA polymerase (TaKaRa Bio Inc., Tokyo, Japan) was used for all the PCR amplifications. All PCR amplification tests were carried out with positive controls (chicken-derived *C. bailey* DNA for *Cryptosporidium* spp. and deer-derived genotype BEB6 DNA for *E. bieneusi*) and negative controls which contained no DNA.

### DNA sequencing and analysis

All nested PCR products were sequenced using the same PCR primers used for the secondary PCRs on an ABI PRISMTM 3730 DNA Analyzer (Applied Biosystems, Carlsbad, CA, USA), using a BigDye Terminator v3.1 Cycle Sequencing kit (Applied Biosystems). The accuracy of the sequencing data was confirmed by sequencing of the PCR products in both directions. Further PCR products were sequenced for some DNA preparations, from which we obtained the sequences with single nucleotide substitutions, deletions or insertions compared to those published in GenBank. The species and genotypes of *Cryptosporidium* and *E. bieneusi* isolates were identified by aligning and analyzing the nucleotide sequences with each other and with reference sequences from GenBank using the Basic Local Alignment Search Tool (BLAST) and Clustal X 1.83 [56]. All the *E. bieneusi* genotypes were named based on 243 bp of the ITS region according to the established nomenclature system [57].

## Results

### Prevalence of *Cryptosporidium* spp. and *E. bieneusi*

Totals of 22 (9.1%) and 19 (7.9%) out of 242 brown rats were found to be infected with *Cryptosporidium* spp. and *E. bieneusi*, respectively. *Cryptosporidium* spp. was found in five areas, with infection rates ranging between 6.7–16.4% except the subdistrict of Harbin City. *Enterocytozoon bieneusi* was found in all the six areas investigated, with infection rates ranging between 1.8–27.0%. No mixed infections of the two pathogens were found in rodents in our study (Table 1).

### *Cryptosporidium* species/genotypes

Sequence analysis of SSU rRNA gene products of 22 *Cryptosporidium* isolates identified four *Cryptosporidium* species and genotypes, including *Cryptosporidium* rat genotype I (14/22, 63.6%), *Cryptosporidium* rat genotype IV (6/22, 27.3%), *Cryptosporidium suis-*like genotype (1/22, 4.5%) and *C. ubiquitum* (1/22, 4.5%). *Cryptosporidium* rat genotype I showed dominance in brown rats in the investigated regions (Table 1).

At the SSU rRNA locus, 14 *Cryptosporidium* rat genotype I isolates had 100% homology between each other and were identical to that (FJ205699) from waste water in China and those from *R. norvegicus* in Sweden (JN172971), *R. rattus* in Iran (KP883289) and raw water in the UK (GQ183517). Of the six sequences of *Cryptosporidium* rat genotype IV, five have not been reported previously. Among them, four sequences were identical to each other (MG917670), with one base substitution at position 309 (C→T) when compared with that from raw water in the UK (GQ183515); the fifth (MG917671) had one base difference compared with that from storm water in the USA (AY737583). The sixth sequence had 100% similarity with those from *R. norvegicus* in Sweden (JN172970) and storm water in the USA (AY737585). The sequences of *C. ubiquitum* and *Cryptosporidium suis-*like isolates obtained here had 100% similarity with those from *Apodemus flavicollis* in Poland (KC962124) and a human in the UK (HQ822146), respectively.

### *Enterocytozoon bieneusi* genotypes

By analyzing nucleotide sequence of the ITS region of the rRNA gene of *E. bieneusi*, we identified two known genotypes D and Peru6 in the brown rats, which had 100% homology with the two sequences GQ406055 and KX375800, respectively. Genotype D was found to be dominant, in 88.9% (17/19) of *E. bieneusi* isolates. Genotype D was also found to have a wide distribution, identified in all regions investigated. The remaining two rodents were infected with genotype Peru6, with 10.5% (2/19) frequency. This genotype was only found in two brown rats captured in the sheep farm (Table 1).

## Discussion

In the present study, 9.1% of the brown rats examined were found to be infected with *Cryptosporidium* spp. The infection rate of *Cryptosporidium* was lower than those reported in brown rats in Iran (17.1%), Japan (38.0%), the Philippines (18.6%) and Sweden (12.0%) [23, 26, 31, 35], but higher than those reported in China (5.6% and 7.1%), Iran (4.1%) and Nigeria (1.5%) [19, 20, 24, 30]. *Cryptosporidium* spp. has been detected in various rodent species. Variable prevalence rates have been observed, such as 8.0–31.4% in mice, 2.1–63.0% in rats and 0.8–73.0% in voles [58], with the lowest and the highest prevalence rates in muskrats (0.7%) and in guinea pigs (85.0%), respectively [19, 59]. In the present study, *E. bieneusi* was detected in brown rats for the first time, with a prevalence of 7.9%. To date, there are only eight studies of *E. bieneusi* infection in rodents worldwide [22, 41–47]. In general, the prevalence of *E. bieneusi* in brown rats here was higher than those in chinchillas (3.6%) and mice (1.1%) [22, 45], but lower than those in hamster family (24.3%), prairie dogs (48.3%), squirrel family (16.7–42.9%), voles (39.1%) and mice (10.5–87.5%) [41–44, 46, 47]. The difference in prevalence may be related to rodent species, detection methods, sample size, animal age and study locations [58].

In the present study, four *Cryptosporidium* species/genotypes were identified including *Cryptosporidium* rat genotype I and IV, *Cryptosporidium suis*-like genotype and *C. ubiquitum*. Previous molecular epidemiological data revealed the presence of at least 11 species and 20 genotypes of *Cryptosporidium* spp. in rodents worldwide [15–38]. In China, five *Cryptosporidium* species (*C. parvum*, *C. muris*, *C. andersoni*, *C. ubiquitum* and *C. wrairi*) and six *Cryptosporidium* genotypes (mouse genotype I, rat genotypes II and III, ferret genotype, chipmunk genotype III and hamster genotype) have been found in rodents [19, 20, 22]. The species and genotype identification of rodent-derived *Cryptosporidium* spp. will be helpful to understand the roles that rodents play in the transmission of cryptosporidiosis.

Several studies have revealed that rats appear to be a major animal host for *Cryptosporidium* rat genotype I and *Cryptosporidium* rat genotype IV. *Cryptosporidium* rat genotype I (previously rat genotype) has been found in brown rats from Philippines, Sweden and Nigeria, and *Cryptosporidium* rat genotype IV (previously W19) in brown rats from Japan and Sweden [26, 30, 31, 35]. Furthermore, the two genotypes have also been detected in environmental samples, including a stream in the USA [48], raw water in the UK and China [60, 61] and the South Nation River watershed in Canada [62]. However, *Cryptosporidium* rat genotype I and *Cryptosporidium* rat genotype IV were found in brown rats in China for the first time. To date, the potential of the two genotypes causing disease in humans or livestock is unknown. In the future, more systematic molecular epidemiological investigations of *Cryptosporidium* spp. in more hosts need to be carried out to understand the true host range of the two genotypes.

*Cryptosporidium ubiquitum*, previously known as the *Cryptosporidium* cervine genotype, infects the largest number of host species of animals [63]. It has been found in domesticated and wild ruminants, a colony of non-human primates (lemurs) and a variety of rodents [22, 29, 32, 35, 38, 63]. To date, human cases of cryptosporidiosis caused by *C. ubiquitum* have been documented in more than ten countries [64]. In addition, this species has also been found in some water bodies, including source water, storm water and raw waste-water [63]. *Cryptosporidium suis*-like genotype has been recorded in rats in Philippines [31] and other animal species including cattle in Denmark, India and China, and yaks in China [65–68]. This genotype was also found in humans in Canada [69]. Although *C. ubiquitum* and *Cryptosporidium suis*-like genotype only accounted for 9.1% of all the *Cryptosporidium* isolates in investigated brown rats, we still need to consider them as a threat to human health, especially *Cryptosporidium* suis-like genotype, for this genotype was found in a granary.

To date, 35 *E. bieneusi* genotypes have been identified in rodents worldwide, 12 of which (BEB6, C, D, EbpA, EbpC, H, Peru 8, Peru11, Peru 16, PigITS5, S6 and TypeIV) have been detected in humans [22, 41–48]. In the present study, two known zoonotic *E. bieneusi* genotypes, D and Peru 6, were identified in brown rats. Genotype D was identified in 88.9% of *E. bieneusi* isolates and was found to have a wide distribution; it was found in all areas investigated. Genotype D is reported to be responsible for most human infections and it has been found in humans from more than 40 countries or areas [1]. It is also isolated in at least 15 species of animals as well as in some water bodies [70, 71]. In the present study Peru6 was only identified in two *E. bieneusi* isolates from two rats captured in the sheep farm. This genotype has been recorded in humans in Peru and Portugal [72–74], and some mammalian animal species and bird species [5, 75]. It has also been detected in wastewater in China [71]. To our knowledge, genotype Peru6 was found in rodents for the first time globally, indicating that this genotype might have more reservoir hosts than expected. The finding of two genotypes previously reported in humans suggests the possibility of rodents in the transmission of *E. bieneusi* to humans [1, 5].

## Conclusions

The present study demonstrated the occurrence of *Cryptosporidium* spp. and *E. bieneusi* in brown rats in Heilongjiang, China and genetically characterized the isolates. We identified *E. bieneusi* genotype Peru6 in rodents for the first time, and *Cryptosporidium* rat genotype I and *Cryptosporidium* rat genotype IV in rats in China for the first time. The finding of zoonotic *C. ubiquitum* and *Cryptosporidium suis*-like genotype as well as two *E. bieneusi* genotypes suggests that brown rats pose a threat to human health. Thus, it is strongly recommended to take measures to control brown rat populations in the areas investigated and improve local people’s awareness of the transmission risk of these two diseases from brown rats to humans.

## Abbreviations

AIDS: Acquired immunodeficiency syndrome
BLAST: Basic Local Alignment Search Tool
EPA: Environmental Protection Agency
HIV: Human immunodeficiency virus
ITS: Internal transcribed spacer
NIH: National Institutes of Health
SSU: Small subunit
